# A Comparative Evaluation of Advanced Platelet-Rich Fibrin Combined With Demineralized Freeze-Dried Bone Allograft and Demineralized Freeze-Dried Bone Allograft Alone in the Treatment of Periodontal Infrabony Defects: A Clinical and Radiographic Study

**DOI:** 10.7759/cureus.61808

**Published:** 2024-06-06

**Authors:** Aakash Goswami, Shivani Lanjewar, Sachin Mangalekar, Vidya Dodwad, Ranu Oza, Priyanka Vhanmane, Unnati Shirbhate

**Affiliations:** 1 Department of Periodontics, Maitri College of Dentistry and Research Center, Durg, IND; 2 Department of Periodontics, Bharati Vidyapeeth Dental College and Hospital, Sangli, IND; 3 Department of Periodontics, Bharati Vidyapeeth Dental College and Hospital, Pune, IND; 4 Department of Periodontics, Sharad Pawar Dental College and Hospital, Datta Meghe Institute of Higher Education and Research, Wardha, IND

**Keywords:** radiographic, osseous defects, periodontal regeneration, infrabony defects, advanced prf, dfdba

## Abstract

Aim

Allografts, autografts, alloplast and xenografts are frequently used for periodontal regeneration. The aim of this study was to determine the efficacy of advanced platelet-rich fibrin (A-PRF) in combination with demineralized freeze-dried bone allograft (DFDBA) and DFDBA alone in periodontal infrabony defects.

Methodology

This was a split-mouth design study where 20 infrabony defects in 10 patients were included. Patients were randomly divided into two groups, where DFDBA allograft and A-PRF were used in the test group, while the DFDBA allograft alone was used in the control group. Furthermore, the results were evaluated at baseline, three, and nine months, respectively, in terms of clinical and radiographic parameters. Data were analysed with an unpaired t-test at the significance level of P < 0.05 (statistically significant).

Results

Both treatments showed reduced clinical and radiographic parameters from baseline to nine months. There was a non-significant difference in the plaque index (PI), bleeding on probing (BOP), clinical attachment level (CAL), and radiographic defect fill (RDF). In comparison to the control group (3.40 ± 0.516), the probing pocket depth (PPD) in the test group at nine months (3.22 ± 0.422) was statistically significant showing reduction in the PPD (P = 0.042).

Conclusion

Within its limitations, the study showed that A-PRF plus DFDBA and DFDBA alone treatment modalities reduced clinical and radiographic parameters from baseline, at 9 months; however, the inclusion of A-PRF did not substantially improve the treatment outcome when comparing both the groups, except for the probing pocket depth after nine months.

## Introduction

Allografts, autografts, alloplast and xenografts are frequently used for periodontal regeneration where the periodontal tissues that have been lost are regenerated, which is the desirable result of periodontal therapy [1]. Periodontal regeneration in autografts and allografts such as demineralized freeze-dried bone allograft (DFDBA) is recognized via histology [2-4]. Due to the restricted amount of bone that is readily available and the potential necessity for a second surgical site, an often less desirable option is to harvest intraoral autogenous bone for periodontal regeneration. During demineralizing, bone morphogenetic protein exposure can induce osteoinductive potential; hence, DFDBA is a frequently used substitute [5-6].

In dentistry, platelet-rich plasma (PRP), platelet-rich fibrin (PRF), and adhesives are administered therapeutically in many ways [7]. Fibrin materials are employed as they imitate the natural coagulation cascade. An innovative concept for cell-based tissue engineering is advanced PRF (A-PRF), initially introduced in 2014 with a minimization of the standard PRF's revolution per minute (RPM) and an increase in its time [8]. The present study was carried out to gain more insights into the clinical efficacy of A-PRF with DFDBA and DFDBA alone when addressing the treatment of periodontal infrabony defects.

## Materials and methods

Study design

The study included 20 sites of infrabony defects from 10 patients selected from the periodontology department of the Bharati Vidyapeeth Dental College and Hospital (Deemed to be University), Sangli, India. These patients were diagnosed with chronic periodontitis and had radiographs that showed a corresponding vertical or angular bone loss in two different quadrants, clinically having two or more infrabony pockets at least 5 mm deep, in either mesial or distal side, and requiring periodontal regenerative therapy to repair the osseous defect. Clinical photographs, clinical parameters and radiographs were obtained before and after the surgery for diagnostic criteria of the defects.

Socio-demographics of the study population

In the present study, 10 patients (six males and four females) in the age range of 25-55 years attending the outpatient department (OPD) of the Department of Periodontology were selected irrespective of caste, religion, socio-economic status, and marital status. Based on the outcome variable on the newly formed bone with a mean difference of 2, 95% statistical power, and 5% significance, a sample size of 10 in each study group was considered adequate for this clinical trial.

Ethical clearance

This study was approved by the Institutional Ethics Committee of Bharati Vidyapeeth (Deemed to be University) Medical College and Hospital, Sangli (BV(DU)MC & H/Sangli/IEC/Dissertation 2018-19/D-06).

Inclusion and exclusion criteria

Patients in the age group of 25-55 years, those with a diagnosis of chronic periodontitis, having clinically two or more infrabony pockets with an at least 5-mm depth with radiographs revealing corresponding vertical bone loss in two different quadrants, and patients willing to complete the study and sign an informed written consent were included. Criteria for exclusion were one wall defect, pregnant and lactating females, smokers and tobacco chewers, grade II and III mobility associated with the respective tooth, history of systemic diseases, and patients exhibiting poor oral hygiene during phase I of the treatment.

Allocation concealment

Before initiating the surgery, 20 infrabony defects were randomly allocated a case number by computer-generated randomization and divided into test and control groups, with 10 in each. In the control group, open flap debridement was used to treat the patient, and only demineralized freeze-dried bone allograft was placed in the defect, while the test group received open flap debridement, and advanced platelet-rich fibrin and DFDBA were placed in infrabony defects. Before initiating the surgical procedure, informed written consent were obtained from all the patients for surgical as well as blood extraction procedures needed for A-PRF preparation.

Preparation of A-PRF

Antecubital vein venipuncture was used for blood extraction; 10 ml of a patient's blood was put in a sterilised test tube without any anticoagulant or other artificial biochemical modifications. A tabletop centrifuge was used to centrifuge the blood right away for 14 minutes at 1500 rpm (SHK Quantos centrifuge system; New Delhi, India). When the centrifugation is performed at low settings for a shorter duration, it is difficult to distinguish between the three layers. The top layer of the straw-coloured liquid, which is abundant in platelets and white blood cells (WBCs), is thus gathered and utilised as the A-PRF clot or compressed to produce an APRF membrane.

Surgical procedure

**Figure 1:**
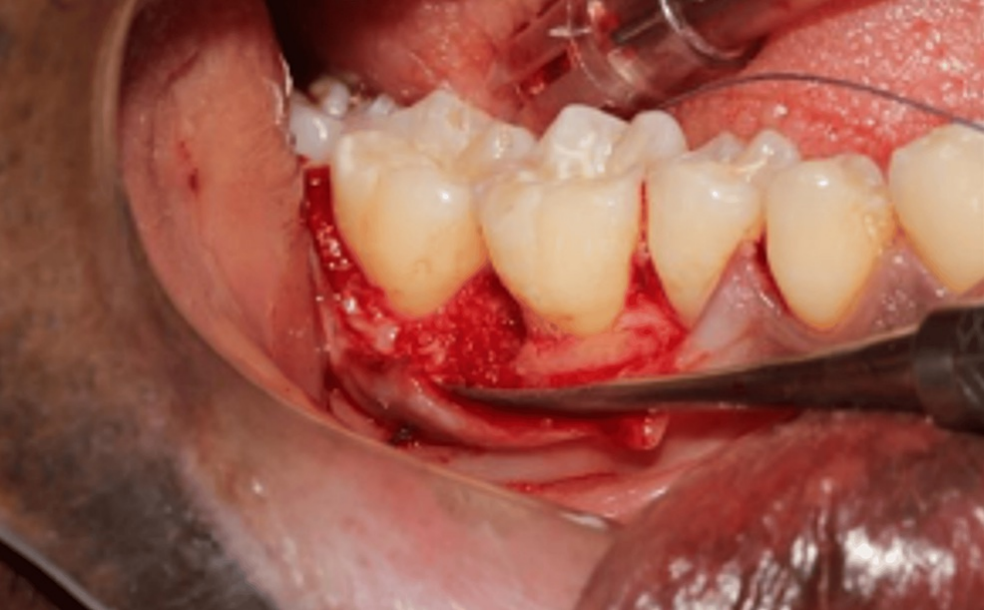
DFDBA placed in the infrabony defect in the control group DFDBA, demineralized freeze-dried bone allograft

**Figure 2:**
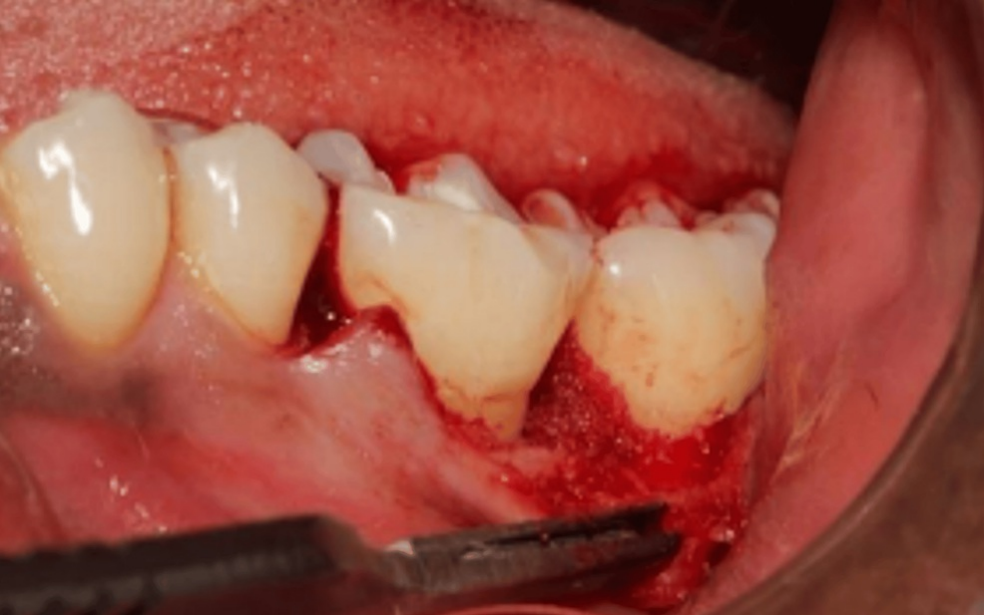
DFDBA and A-PRF placed in the infrabony defect in the test group DFDBA, demineralized freeze-dried bone allograft; A-PRF, advanced platelet-rich fibrin

Initially the scaling and root planing (SRP) was performed and after six weeks of initial therapy, the patient was taken for surgery, considering the inclusion and exclusion criteria. Under all aspetic conditions and precautions and under local anaesthesia, the full-thickness mucoperiosteal flap with respective osseous defect was raised and debridement was carried out; the test group received A-PRF and DFDBA and the control group received DFDBA (Figures 1-2). Haemostasis was achieved and sutures were placed. A periodontal pack given and the patient was recalled after seven days for suture removal; post-operative instructions were also given. The patient was reviewed for three, six and nine months, respectively, and clinical and radiographic examinations were carried out for getting clinical outcomes of the study.

Statistical analysis

Descriptive statistic analysis was applied to assess means, standard deviations, and frequencies using SPSS Statistics for Windows, version 17 (SPSS Inc., Chicago, IL). An unpaired t-test was done for the inter-group comparison and a repeated measures ANOVA test for the within-group comparison.

## Results

Comparisons between the test and control groups were done using the Student's unpaired test. The results for both groups were obtained in terms of the plaque index (PI), bleeding on probing (BOP), probing pocket depth (PPD), clinical attachment level (CAL), and radiographic defect fill (RDF).

The values obtained for the PI and BOP at baseline, and three, six, and nine months for both groups are shown in Table 1. An unpaired t-test was performed on each of them. In the test group, the reduction was 2.50 ± 0.527 (PI) and 2.80 ± 0.422 (BOP) to 1.20 ± 0.422 (PI) and 0.40 ± 0.516 (BOP) following surgery. At the third, sixth, and ninth month following surgery, the control group experienced a decrease, from 2.50 ± 0.527 (PI) and 2.80 ± 0.422 (BOP) to 1.30 ± 0.483 (PI) and 0.80 ± 0.422 (BOP), correspondingly. There was no statistically significant difference in the PI between the experimental and control groups. However, the bleeding index's significant P-value suggests that the DFDBA + A-PRF combination was successful in lowering the proportion of the bleeding index at six and nine months.

**Table 1:** Plaque index (PI) and bleeding on probing (BOP) comparison at baseline, three, six, and nine months between both groups ^a^Silness and Löe plaque index ^b^Ainamo and Bay gingival bleeding index *Statistically significant difference

		PI^a^			BOP^b^		
	Group	Mean	Std. deviation	P-value	Mean	Std. deviation	P-value
Baseline	Test group	2.50	0.527	1.000	2.80	0.422	1.00
	Control group	2.50	0.527		2.80	0.422	
Three months	Test group	1.20	0.422	1.000	0.70	0.483	0.398
	Control group	1.20	0.422		0.60	0.516	
Six months	Test group	1.20	0.422	1.000	0.50	0.527	0.001*
	Control group	1.20	0.422		0.90	0.316	
Nine months	Test group	1.20	0.422	1.000	0.40	0.516	0.04*
	Control group	1.30	0.483		0.80	0.422	

Table 2 shows the values obtained for the probing pocket depth and clinical attachment level at baseline, three, six, and nine months in both groups. At three, six, and nine months after surgery, the test group's mean reduction in probing depths was 3.80 ± 0.632, 2.90 ± 0.316, and 3.20 ± 0.422, respectively. At three, six, and nine months after surgery, the control group's decrease was 4.20 ± 0.422, 3.30 ± 0.483, and 3.40 ± 0.516, respectively. The difference in these metrics between the experimental and control groups was not statistically significant; however, by nine months, it was highly significant (P =0.042). The intragroup comparison of the clinical attachment level between the two groups at baseline (P = 1.00), three (P = 1.00), six (P = 1.00), and nine months (P = 0.177) was not significant.

**Table 2:** Probing pocket depth (PPD) and clinical attachment level (CAL) comparison at baseline, three, six, and nine months between both groups *Statistically significant difference

		PPD			CAL		
	Group	Mean	Std. deviation	P-value	Mean	Std. deviation	P-value
Baseline	Test group	9.10	1.101	0.628	9.30	0.949	1.000
	Control group	9.00	1.054		9.30	1.160	
Three months	Test group	3.80	0.632	.838	4.20	0.422	1.000
	Control group	4.20	0.422		4.20	0.422	
Six months	Test group	2.90	0.316	0.113	3.20	0.422	1.000
	Control group	3.30	0.483		3.20	0.422	
Nine months	Test group	3.20	0.422	.042*	3.20	0.422	0.177
	Control group	3.40	0.516		3.50	0.527	

Table 3 shows the values obtained for the radiographic defect fill in the control and test groups at baseline, three, six, and nine months. While analyzing the data taken at baseline (P = 0.601), three months (P = 1.00), six months (P = 0.361), and nine months (P = 0.177), there was no statistically significant change in the radiographic bone level within the groups. The intra-group comparison of the radiographic defect fill in the control group was reduced from baseline to nine months; the sites treated with DFDBA showed a statistically significant reduction in the radiographic defect fill. Similarly, a statistically significant radiographic defect fill was observed at the sites treated with DFDBA + A-PRF in the intra-group comparison between baseline and the nine-month postoperative follow-up.

**Table 3:** Comparison of the radiographic defect fill (RDF) between the control and test groups at baseline, three, six, and nine months

	Group	Mean	Std. deviation	P-value
Baseline	Test group	11.10	1.101	.601
	Control group	10.80	1.398	
Three months	Test group	9.90	1.197	1.00
	Control group	9.90	1.449	
Six months	Test group	7.40	0.843	0.361
	Control group	7.00	1.054	
Nine months	Test group	5.10	0.568	0.177
	Control group	5.70	0.675	

**Figure 3:**
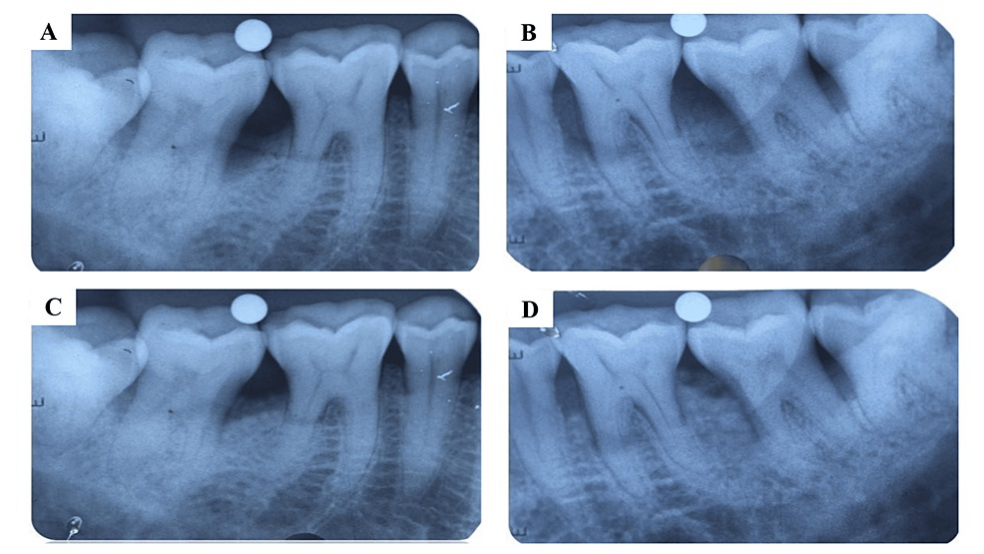
Radiographic defect fill: (A) a preoperative radiograph of the defect treated with DFDBA, (B) a preoperative radiograph of the defect treated with DFDBA mixed with A-PRF, (C) a postoperative radiograph of the defect treated with DFDBA, (D) a postoperative radiograph of the defect treated with DFDBA mixed with A-PRF DFDBA, demineralized freeze-dried bone allograft; A-PRF, advanced platelet-rich fibrin

A radiographic defect fill in both the groups shown by the intraoperative radiograph taken preoperatively and postoperatively was appreciated (Figure 3).

## Discussion

DFDBA serves as both an osteoinductive factor and the source of the osteoconductive surface. In the present study, we used DFDBA of <500 um particulate size, from the TATA Memorial Tissue Bank, Mumbai. The addictive effect of A-PRF on DFDBA was tested; previous studies have shown that PRP, concentrated growth factors, and leukocyte-platelet-rich fibrin are inferior to A-PRF in terms of growth factor release, making it one of the most regenerative PRF subtypes due to the maximum concentration of WBCs and platelet release [9,10].

The current study used the split-mouth design to choose 20 sites in 10 patients randomly. This minimised the impact of interpatient variability while excluding the influence of patient-specific characteristics, making trial interpretation easier. Because no re-entry surgery was planned at postoperative visits due to ethical concerns and associated patient resistance, clinical and radiographic criteria were necessary to standardise the follow-up method. To prevent variations in the tissue response due to the age factor, patients under 25 and those over 55 were excluded from the study. Additionally, individuals with systemic conditions like diabetes mellitus or those taking immunosuppressive medications were eliminated from this study because wound healing is almost always compromised in these patients.

The custom-made stents with vertical grooves offer support and guidance to maintain the probe's direction while analysing clinical recordings. A 0.5 ball bearing was used in the stent to uniformly angle intraoral periapical (IOPA). The occlusal stents were left intact in the study model throughout the investigation to prevent distortion. A fixed distance was maintained between the cone and the X-ray films as intraoral periapical radiographs were obtained at baseline, three, six, and nine months after treatment to evaluate the alveolar bone changes. Using ImageJ software (https://imagej.net/ij/), the radiographic measurements were captured. The linear distance from 0.5 to the defect’s base was calculated to assess the defect fill and change in the alveolar crest height.

Patients were advised to follow strict plaque control measures, and thorough oral prophylaxis was performed three months and six months after surgery, as the benefits of plaque control are well accepted. Systemic antibiotics were prescribed if necessary to reduce any postoperative infection.

When comparing the plaque index among the test and control groups, no statistically significant difference was observed during the baseline, three, six, and nine months. The variation between the test and control groups at three months from the baseline concerning the bleeding index difference was not statistically significant. Still, at six and nine months, it was highly significant. A similar clinical trial by Kukreja et al. revealed that the differences in bleeding and plaque indexes were not statistically significant [11]. According to Gurinsky et al., for both groups, PPD values were statistically insignificant at baseline, and three- and six-month periods but were highly significant at nine months. The intergroup evaluation between the two groups for clinical attachment level gain was not statistically significant at baseline, and three, six, and nine months in terms of clinical and radiographic parameters except for PPD that was statistically significant [12]. In contrast to the study mentioned above, results of a meta-analysis done by Xue et al. showed that DFDBA in conjunction with rich platelet derivatives outperformed DFDBA or rich platelet derivatives alone [13].

The intergroup comparison between the test and control groups concerning the radiographic defect fill was not statistically significant at baseline, three, six, and nine months. These findings were based on a study by Khosropanah et al.; however, a significant difference was observed in contrast to the above-mentioned study by Bansal et al. [14,15]. Both treatments showed an extremely substantial reduction in the plaque index, gingival bleeding index, clinical attachment level, radiographic defect fill, and probing pocket depth from baseline to nine months. The addition of A-PRF to DFDBA did not significantly enhance clinical measures, except for improving probing pocket depth at nine months. The study's shortcomings were a limited sample size, a brief follow-up period, a need for re-entry, and a non-digital radiography examination.

## Conclusions

A substantial decrease in the radiographic defect fill was noted in both groups over the study period, with more reduction noted in the DFDBA + A-PRF group (5.10 ± 0.568). Except for the probing pocket depth, which indicated a substantial decrease in the PPD at nine months in the test group, the entire sites treated with DFDBA and DFDBA + A-PRF showed no notable variations between the two groups in any metrics. This clinical trial suggests the safe and useful application of A-PRF in infrabony defects. More research and clinical studies should be carried out on the application of A-PRF with other bone graft materials in periodontal defect regeneration in larger sample sizes.
